# P53 and Cancer-Associated Sialylated Glycans Are Surrogate Markers of Cancerization of the Bladder Associated with *Schistosoma haematobium* Infection

**DOI:** 10.1371/journal.pntd.0003329

**Published:** 2014-12-11

**Authors:** Júlio Santos, Elisabete Fernandes, José Alexandre Ferreira, Luís Lima, Ana Tavares, Andreia Peixoto, Beatriz Parreira, José Manuel Correia da Costa, Paul J. Brindley, Carlos Lopes, Lúcio L. Santos

**Affiliations:** 1 Experimental Pathology and Therapeutics group, Portuguese Institute for Oncology of Porto, Porto, Portugal; 2 Clínica Sagrada Esperança, Luanda, Angola; 3 Grupo de Investigação em Cancro Digestivo (GICD), Porto, Portugal; 4 Department of Chemistry of the University of Aveiro, Aveiro, Portugal; 5 Research Department, LPCC-Portuguese League Against Cancer (NRNorte), Porto, Portugal; 6 Núcleo de Investigação em Farmácia – Centro de Investigação em Saúde e Ambiente (CISA), School of Allied Health Sciences – Polytechnic Institute of Porto, Porto, Portugal; 7 Department of Pathology, Portuguese Institute for Oncology of Porto, Porto, Portugal; 8 Center for the Study of Animal Science (ICETA), University of Porto, Porto, Portugal; 9 INSA, National Institute of Health, Porto, Portugal; 10 Research Center for Neglected Diseases of Poverty- Department of Microbiology, Immunology & Tropical Medicine, School of Medicine & Health Sciences, George Washington University, Washington, D.C., United States of America; 11 Abel Salazar Biomedical Sciences Institute (ICBAS), University of Porto, Porto, Portugal; 12 Health School of University of Fernando Pessoa, Porto, Portugal; 13 Department of Surgical Oncology, Portuguese Institute for Oncology, Porto, Portugal; 14 National Cancer Center, Luanda, Angol; Queensland Institute of Medical Research, Australia

## Abstract

**Background:**

Bladder cancer is a significant health problem in rural areas of Africa and the Middle East where *Schistosoma haematobium* is prevalent, supporting an association between malignant transformation and infection by this blood fluke. Nevertheless, the molecular mechanisms linking these events are poorly understood. Bladder cancers in infected populations are generally diagnosed at a late stage since there is a lack of non-invasive diagnostic tools, hence enforcing the need for early carcinogenesis markers.

**Methodology/Principal Findings:**

Forty-three formalin-fixed paraffin-embedded bladder biopsies of *S. haematobium*-infected patients, consisting of bladder tumours, tumour adjacent mucosa and pre-malignant/malignant urothelial lesions, were screened for bladder cancer biomarkers. These included the oncoprotein p53, the tumour proliferation rate (Ki-67>17%), cell-surface cancer-associated glycan sialyl-Tn (sTn) and sialyl-Lewis^a/x^ (sLe^a^/sLe^x^), involved in immune escape and metastasis. Bladder tumours of non-*S. haematobium* etiology and normal urothelium were used as controls. *S. haematobium*-associated benign/pre-malignant lesions present alterations in p53 and sLe^x^ that were also found in bladder tumors. Similar results were observed in non-*S. haematobium* associated tumours, irrespectively of their histological nature, denoting some common molecular pathways. In addition, most benign/pre-malignant lesions also expressed sLe^a^. However, proliferative phenotypes were more prevalent in lesions adjacent to bladder tumors while sLe^a^ was characteristic of sole benign/pre-malignant lesions, suggesting it may be a biomarker of early carcionogenesis associated with the parasite. A correlation was observed between the frequency of the biomarkers in the tumor and adjacent mucosa, with the exception of Ki-67. Most *S. haematobium* eggs embedded in the urothelium were also positive for sLe^a^ and sLe^x^. Reinforcing the pathologic nature of the studied biomarkers, none was observed in the healthy urothelium.

**Conclusion/Significance:**

This preliminary study suggests that p53 and sialylated glycans are surrogate biomarkers of bladder cancerization associated with *S. haematobium*, highlighting a missing link between infection and cancer development. Eggs of *S. haematobium* express sLe^a^ and sLe^x^ antigens in mimicry of human leukocytes glycosylation, which may play a role in the colonization and disease dissemination. These observations may help the early identification of infected patients at a higher risk of developing bladder cancer and guide the future development of non-invasive diagnostic tests.

## Author Summary

Epidemiological studies associate infection with *S. haematobium*, an endemic parasitic flatworm in Africa and the Middle East, with the development of bladder cancer. Nevertheless, little molecular evidence exists supporting this association. This work draws attention to the common molecular pathways underlying these two events, highlighting a potentially unreported link between infection and cancer development. It has been demonstrated that a panel of biomarkers commonly associated with aggressive forms of bladder cancer is also present in non-malignant tissues infected with the parasite. This may offer a means of early identification of people with this parasitic infection who are at risk of developing of bladder cancer, and may guide the establishment of non-invasive diagnostic tests. Furthermore, we observed that parasite eggs mimic the molecular nature of human cells, providing a possible mechanism of immune escape and persistent infection. Such knowledge is considered pivotal to develop novel therapeutic strategies.

## Introduction

*Schistosoma haematobium*, a parasitic flatworm infecting millions of people in Angola and other countries of Africa and Middle East, is responsible for the development of urinary schistosomiasis, a neglected tropical disease [1], [2]. The World Health Organization estimates that 500 to 600 million people residing in rural agricultural and periurban areas are at risk of infection and over 200 million people are currently infected, 10% of which will experience sever health complications; [3], [4].

The parasite has a complex life cycle consisting of two phases, one inside the human body (the definitive host) and another inside a snail of the genus *Bulinus* [5]. Free-swimming cercariae penetrate human skin when in contact with contaminated water, enter the blood stream and travel to the liver to mature into adult flukes. After a period of about three weeks the young flukes migrate to the plexuses around the urinary bladder to copulate. The eggs released by female flukes traverse the wall of the bladder causing haematuria, fibrosis and ultimately the calcification of the tissue; they are then excreted through urine [6], [7]. However, some eggs become embedded in the bladder mucosa further contributing to chronic inflammation and granuloma formation [6], [7]. The eruption of the eggs through the mucosa stimulates not only the establishment of chronic inflammations but also promotes the development of benign/pre-malignant bladder lesions such as urothelial hyperplasia and dysplasia that may be precursors of bladder cancer [8]–[10]. When contaminated urine comes in contact with fresh watercourses (e.g. rivers), the eggs hatch, releasing free-swimming miracidia that infect the intermediate snail host. After a maturation period new cercariae are formed and released into the environment, assuring the perpetuation of infection and transmission of the disease [5].

The World Health Organization (WHO) International Agency for Cancer classifies *S. haematobium* as a Group 1 biological carcinogen, a definitive cause of cancer [11]. Epidemiological findings reveal a positive relationship between *S. haematobium* infection and the development of squamous cell carcinoma of the bladder, a type of bladder cancer rarely observed in western patients but prevalent in Africa and Middle East [12]–[14]. It has been observed that patients infected with the parasite have a higher risk of developing bladder cancer earlier in life than uninfected people [13], [15]. The probability of developing cancer has been suggested to depend on the intensity (worm burden and tissue egg burden) and duration of infection [16], [17]. However, despite the epidemiological data from case control studies and the geographical overlap between bladder cancer development and regions endemic for urogenital schistosomiasis [8], [18], few experimental evidences support this association. Nonetheless, Botelho and coworkers demonstrated recently that the exposure to soluble antigen extracts of mixed sex adult *S. haematobium* worms and eggs promote the tumourogenic potential of urothelial cells in *vitro* and *in vivo* [8], [19], [20], and Zhong and colleagues have reported hypermethylation of several genes including *RASSF1A* and *TIMP3* detected in urine sediments of Ghanaians with bladder pathology associated with infection with *S. haematobium* [21].Further understanding of the pathobiological features underlying the association between *S. haematobium* and bladder cancer development are needed to support these observations. The identification of the molecular events underlying early urothelial carcinogenesis in the bladder is also warranted. This is a particular critical matter since early symptoms of schistosomiasis, which include urinary pain and hematuria, are common to those of bladder cancer. As such, they are often neglected by local communities in developing countries, where medical assistance is scarce. Therefore, bladder tumours are often diagnosed at a late stage, which is associated with decreased overall survival. The identification of biomarkers may help to control *S. haematobium*-associated bladder cancer in these populations.

This research is based on establishing common molecular alterations among schistosoma-associated tumours and benign/pre-malignant lesions found either in tumor-adjacent mucosa or in apparently normal urothelia of cases without tumors. These lesions were screened for oncoprotein p53 that is associated with both aggressive urothelial [22]–[24] and squamous cell bladder carcinomas [25]. The proliferation rate, given by the overexpression of nuclear protein Ki-67, and considered a prognostic marker of tumor recurrence and progression in non-muscle invasive urothelial carcinoma [26]–[28], was also evaluated. Particular attention was further devoted to the characterization of alterations in membrane-bound glycans that accompanied malignant transformations and favor cell-to-cell detachment, migration, immune evasion and metastization [29]. This includes the sialylated antigens sialyl-Tn (sTn; CA72-4) [30], [31], sialyl-Le^a^ (sLe^a^, CA19-9) [32]–[34] and sialyl-Le^x^ (sLe^x^) [35], [36] that have been observed in bladder cancer. Cancer-associated glycans can also be found in secreted proteins often shed into the bloodstream and urine, offering potential for non-invasive diagnosis [34], [36]–[38].

## Materials and Methods

### Ethics Statement

All procedures were performed after patient's written informed consent and parental consent in the cases of children and approved by the Ethics Committee of Agostinho Neto University, Luanda, Angola and the Portuguese Institute For Oncology of Porto, Portugal (IPO-Porto). Clinico-pathological information was obtained from patients' clinical records and this information was anonymized.

### Cases

This study includes 43 Angolan patients (30.2% male and 69.8% female) diagnosed as positive for *S. haematobium* infection in Sagrada Esperança Clinic (Luanda, Angola) and Hospital Américo Boavida (Luanda, Angola). The median age of the patients was 33.5 years (12–82 years) and, even though the majority resided in the rural areas around Luanda, they were born and had resided in provinces where *S. haematobium* is endemic. All patients presented irregularities of inner surface of bladder wall found by ultrasound scan and some of them showed a localized thickening of bladder wall protruding into the lumen. Therefore, the patients underwent cystoscopy and biopsy of the visualized mass and corresponding adjacent mucosa. The apparently normal urothelium of cases without noticeable tumour mass were also subjected to random biopsies. All biopsies of apparently normal urothelium and tumour-adjacent mucosa presented benign/pre-malignant lesions (chronic inflammation, urothelial hyperplasia, epidermoid metaplasia or dysplasia). Malignant lesions included papilloma (P), papillary urothelial neoplasm of low malignant potential (PUNLMP) and high-grade urothelial cell carcinoma (UCC), squamous cell carcinomas (SCC) or both (UCC+SCC) as summarized in Table 1. No differences were observed in age and sex distribution among the lesions/tumours. *S. haematobium* eggs were evident in the bladder of 27 (62.8%) cases, from these 7 (26%) presented tumours.

**Table 1:** Pathological characterization of the samples.

Benign/Pre-malignant lesions	No tumour (n = 24)	With Tumour (n = 19)				
		P	PTLMP	UC	SCC	UC+SCC
Chronic Inflammation	6	1	-	4	-	1
Urothelial Hyperplasia	8	-	2	-	2	1
Epidermoid Metaplasia	7	-	-	-	5	2
Displasia	3	-	-	-	-	-

P: papilloma; PTLMP: papillary tumour of low malignant potential; UC: urothelial tumour; SCC: squamous cell carcinomas; UC+SCC: urothelial cancer and squamous cell carcinoma.

This study also includes a retrospective series of 22 non-*Schistosoma haematobium* infected patients diagnosed with urothelial cell carcinoma (10 low-grade tumours; 12 high-grade tumours, 5 presenting muscle invasion) and 4 squamous cell carcinomas presenting invasion of the *muscularis propria*, that have been previously characterized in relation to Ki-67 and sTn expressions by Ferreira *et al.* [31]. The patients (48.3% male and 51.7% female), mean age 69 years (45-89 years), underwent transurethral resection of the tumour in the Portuguese Institute for Oncology of Porto (IPO-Porto, Portugal), between July 2011 and May 2012. None had received prior adjuvant therapy. Six normal urothelium tissues of necropsied male individuals without bladder cancer history, within the same mean of age range, were also included.

Formalin fixed paraffin embedded biopsies and tumour sections stained with hematoxylin and eosin were examined and classified by an experienced pathologist under light microscopy, with reference to the WHO's 2004 grading criteria [39].

### Immunohistochemistry

Formalin-fixed, paraffin-embedded (FFPE) tissue sections were screened for p53 accumulation, proliferation (Ki-67), and cancer-associated glycans sTn, sLe^a^, and sLe^x^ by immunohistochemistry by the streptavidin/biotin peroxidase method using mouse monoclonal antibodies. The p53 protein was determined with clone DO-7 (Dako), Ki-67 with clone MIB-1 (Dako), sTn with clone TKH2 [31], sLe^a^ with clone (Abcam) and sLe^x^ with clone (Abcam). Briefly, 3 µm sections were deparaffinized with xylene, rehydrated with graded ethanol series, microwaved for 15 min in boiling citrate buffer (10 mM citric acid, 0.05% Tween 20, pH 6.0), and exposed to 3% hydrogen peroxide in methanol for 20 min. After blockage with BSA (5% in PBS), the antigens were identified with UltraVision Detection System (Thermo Scientific) followed by incubation with 3,3-diaminobenzidine tetrahydrochloride (Impact Dab, Vector). Finally, the slides were counterstained with haematoxylin for 1 min. Colon carcinoma, tonsil and intestinal metaplasia tissue sections were tested in parallel as positive controls for, p53, Ki-67 and sialylated glycans, respectively. Negative control sections were included, involving sections probed with BSA (5% in PBS) devoid of primary antibody. The tissues were also treated with a neuraminidase from *Clostridium perfringens* (Sigma-Aldrich) to remove the sialic acid from the glycans and screened thereafter for sTn, sLe^a^, and sLe^x^, as described by Ferreira et al. [31].

A semi-quantitative approach was established to score the immunohistochemical labeling based on the intensity of staining and the percentage of cells that stained positively. The immunoexpression was assessed blindly by two independent observers and validated by an experienced pathologist. Whenever there was a disagreement, the slides were reviewed, and consensus was reached. Tumours were classified as p53 positive whenever expression was higher than 5% of the tissue section, as proliferative whenever Ki-67 expression was higher than 17%, as described by Santos et al. [26], and sTn, sLe^a^ and sLe^x^ were considered positive whenever the percentage of staining was ≥5% of the tissue sections [31], [32], [35].

### Statistics

Statistical data analysis was performed using the IBM Statistical Package for Social Sciences—SPSS for Windows (version 20.0). Chi-square analysis was used to compare categorical variables. Correlation between cancer associated markers expression in pre-malignant lesions and concomitant tumours whenever present was performed using Pearson correlation test. A *P* value of ≤0.05 was considered to be statistically significant.

## Results

**Figure 1:**
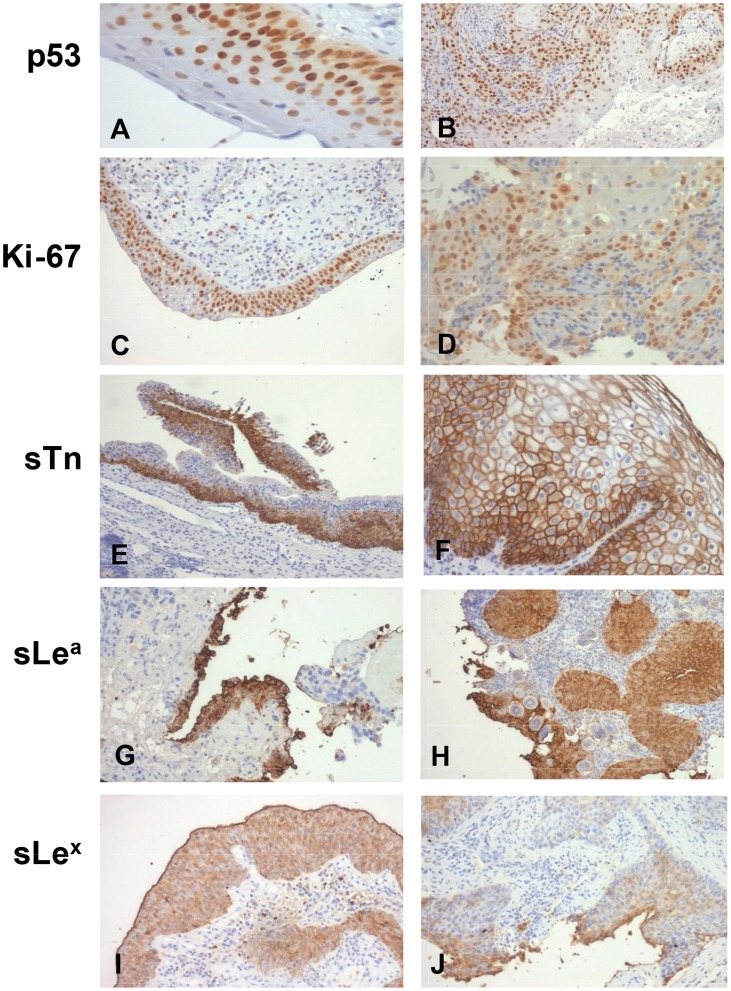
Expression of cancer-associated biomarkers p53, Ki-67, sTn, sLe^a^ and sLe^x^ in bladder benign/pre-malignant lesions. A) Chronic inflammation; B) SCC; C) Chronic inflammation; D) SCC; E) Urothelial hyperplasia; F) Urothelial carcinoma; G) Dysplasia; H) SCC; I) Dysplasia; J) Urothelial carcinoma.

Bladder tumours associated with *S. haematobium* infection were screened for the accumulation of p53, proliferation rate (Ki-67>17%) and cancer-associated sialylated glycans sTn, sLe^a^ and sLe^x^ (Fig. 1). We could observe that all the biomarkers were expressed throughout the different layers of the urothelium in benign/pre-malignant lesions and also homogeneously expressed in the tumours, irrespectively of their histological classification. As presented in Table 2, the majority of the bladder tumors exhibited p53 alterations (84%) and sLe^x^ overexpression (74%). Similar percentages of p53 and sLe^x^ could also be observed in bladder tumour sections from patients non-infected with *Schistosoma haematobium*, irrespectively of their histological natures. Conversely, non-proliferative phenotypes predominated among low malignant lesions (papilloma and PUNLMP; 100% of the cases) when compared to the other groups comprehending more aggressive lesions presenting either invasion and/or high potential to invade the bladder wall (UCC, SCC, SCC+UC; approximately 50% of the cases) (Table 2). Contrasting with these findings, the percentage of proliferative phenotypes in the less aggressive non-schistosome associated lesions (low grade papillary tumours) was 30%. The percentage of proliferative phenotypes in more aggressive high grade lesions, including high grade urothelial cell and squamous cell carcinomas, was similar to described for *S. haematobium* infection related lesions. The sLe^a^ antigen was detected in approximately 50% of the *S. haematobium*-associated malignant lesions, irrespectively of their histology. These observations contrasted with the significantly higher expression of sLe^a^ observed in lesions not associated with the parasite (80% of the cases). Regarding the sTn antigen, its frequency varies among the histological groups of tumours, papilloma and UCC did not express the antigen, PUNLMP and SCC showed an equal distribution of negative and positive cases. However, the antigen was expressed by 75% of the cases presenting both an UCC and SCC phenotype, which is in accordance with our previous results for non-schistosome associated bladder tumors, where sTn antigen was present in approximately 75% of aggressive bladder tumors (high grade papillary tumors and muscle invasive bladder cancer) [31]. Altogether, the studied biomarkers, with the exception of sLe^a^ presented similar expressions in both schistosome and non-schistosome associated bladder tumors.

**Table 2:** Cancer associated markers expression in *Schistosoma haematobium*-associated bladder tumours.

Variables	Papilloma	PUNLMP	UCC	SCC	UCC+SCC	Total
	n (%)	n (%)	n (%)	n(%)	n(%)	n (%)
**p53**						
** Negative**	0 (0.0)	0 (0.0)	0 (0.0)	2 (28.6)	1 (25.0)	3 (15.8)
** Positive (altered)**	1 (100.0)	2 (100.0)	5 (100.0)	5 (71.4)	3 (75.0)	16 (84.2)
**Ki-67**						
** Non proliferative**	1 (100.0)	2 (100.0)	2 (40.0)	3 (42.9)	2 (50.0)	10 (52.6)
** Proliferative (>17%)**	0 (0.0)	0 (0.0)	3 (60.0)	4 (57.1)	2 (50.0)	9 (47.4)
**sTn**						
** Negative**	1 (100.0)	1 (50.0)	5 (100.0)	4 (57.1)	1 (25.0)	12 (63.2)
** Positive**	0 (0.0)	1 (50.0)	0 (0.0)	3 (42.9)	3 (75.0)	7 (36.8)
**sLe^a^**						
** Negative**	-	1 (50.0)	2 (40.0)	4 (57.1)	2 (50.0)	10 (52.6)
** Positive**	-	1 (50.0)	3 (60.0)	3 (42.9)	2 (50.0)	9 (47.4)
**sLe^x^**						
** Negative**	0 (0.0)	0 (0.0)	1 (20.0)	1 (16.7)	2 (50.0)	4 (22.2)
** Positive**	1 (100.0)	2 (100.0)	4 (80.0)	5 (83.3)	2 (50.0)	14 (77.8)

PUNLMP: Papillary Urothelial Neoplasm of Low Malignant Potential.

UCC: Urothelial Cell Carcinoma.

SCC: Squamous Cell Carcinoma.

Benign/pre-malignant lesions associated with *S. haematobium* infection, irrespective of having been isolated from urothelium without malignant lesions or tumour adjacent mucosa, were also studied (Fig. 1). The p53 protein was altered in the majority of the benign/pre-malignant lesions (88%), predominantly in those showing cellular alterations (urothelial hyperplasia, epidermoid metaplasia, dysplasia; Table 3). Approximately 40% of the cases also presented a high proliferation index (Ki-67>17%), although this was more pronounced among epidermoid metaplasia (64.3%, Table 3). The sTn antigen was detected in one third of the lesions (32.6%), however it was mainly absent in chronic inflammation cases and when compared with all the others a trend association was observed (*P* = 0.067). The sialylated Lewis antigens sLe^a^ and sLe^x^ were detected in the majority of the cases (>80%). However, the percentage of sLe^a^ positive cases was higher among the cases with urothelial hyperplasia while sLe^x^ was present in all dysplasia cases (4/4; Table 3). Furthermore, none of the normal urothelium tissues were positive for the studied biomarkers, demonstrating its cancer-associated nature.

**Table 3:** Cancer associated markers expression in benign/pre-malignant lesions.

Variables	Chronic Inflammation	Urothelial Hyperplasia	Epidermoid Metaplasia	Dysplasia	Total
	n (%)	n (%)	n (%)	n(%)	n (%)
**p53**					
** Negative**	3 (25.0)	1 (7.7)	1 (7.7)	0 (0.0)	5 (11.9)
** Positive (altered)**	9 (75.0)	12 (92.3)	12 (92.3)	4 (100.0)	37 (88.1)
**Ki-67**					
** Non proliferative**	6 (54.5)	11 (84.6)	5 (35.7)	3 (75.0)	25 (59.5)
** Proliferative (>17%)**	5 (45.5)	2 (15.4)	9 (64.3)	1 (25.0)	17 (40.5)
**sTn**					
** Negative**	11(91.7)	8 (61.5)	8 (57.1)	2 (50.0)	29 (67.4)
** Positive**	1 (8.3)	5 (38.5)	6 (42.9)	2 (50.0)	14 (32.6)
**sLe^a^**					
** Negative**	4 (33.3)	1 (7.7)	3 (21.4)	1 (25.0)	8 (19.0)
** Positive**	8 (67.7)	12 (92.3)	11 (78.6)	3 (75.0)	34 (81.0)
**sLe^x^**					
** Negative**	3 (25.0)	2 (15.4)	3 (21.4)	0 (0.0)	8 (18.6)
** Positive**	12 (75.0)	11 (84.6)	11 (78.6)	4 (100.0)	35 (81.4)

Table 4 further highlights the relationship between the studied markers in the benign/pre-malignant lesions identified in apparently normal bladder mucosa and those found in tumor adjacent mucosa. The distribution of the p53 alterations, sTn and sLe^x^ antigens overexpression was similar between the two groups. However, a higher number of non-proliferative cases were observed in sole benign/pre-malignant lesions when compared with the lesions in tumour adjacent mucosa (73.9% vs 42.1%, *P* = 0.037; Table 4). On the other hand, sLe^a^ expression was more frequent in lesions without tumour than with concomitant tumours (91.7% vs 66.7%; *P* = 0.03). Altogether this data shows that the majority of the benign/pre-malignant lesions associated with *S. haematobium* infection share alterations in p53 expression and sLe^x^ with bladder tumors. The predominance of sLe^a^ in pre-malignant lesions, in particular in bladders that do not present signs of malignant transformation, suggesting that this glycan may be a molecular alteration associated with early carcinogenesis pathways.

**Table 4:** Relation between cancer associated markers in benign/pre-malignant lesions as sole lesions and in tumour adjacent mucosa.

Variables	Benign/Pre-malignant		
	As sole lesion	In tumour adjacent mucosa	p*
	n (%)	n (%)	
**p53**			
** Negative**	1 (4.2)	4 (22.2)	
** Positive (altered)**	23 (95.8)	14 (77.8)	0.146
**Ki-67**			
** Non proliferative**	17 (73.9)	8 (42.1)	
** Proliferative (>17%)**	6 (26.1)	11 (57.9)	0.037
**sTn**			
** Negative**	15 (62.5)	14 (73.7)	
** Positive**	9 (37.5)	5 (26.3)	0.437
**sLe^a^**			
** Negative**	2 (8.3)	7 (36.8)	
** Positive**	22 (91.7)	12 (63.2)	0.030
**sLe^x^**			
** Negative**	4 (16.7)	4 (21.1)	
** Positive**	20 (83.3)	15 (78.9)	0.714

^*^: Chi-square test;

Table 5 further shows the correlation between the expression of the biomarkers in the tumours and adjacent mucosa lesions. This showed a correlation between the expression of p53, sTn, sLe^a^ and sLe^x^ both in the lesion and tumour, denoting that the tumour adjacent mucosa reflects the molecular alterations found in S. *haematobium*-associated tumours.

**Table 5:** Correlation between cancer associated marker expression in the lesions and in the concomitant tumour.

	Bladder Cancer	
Benign/Pre-malignant Lesions	p53		Ki-67		sTn		sLe^a^		sLe^x^	
	**Correlation coefficient**	*****P** value***	**Correlation Coefficient**	***P* value**	**Correlation Coefficient**	***P* value**	**Correlation Coefficient**	***P* value**	**Correlation Coefficient**	***P* value**
**p53**	0,484	**0,036**	–0,060	0,814	0,033	0,896	0,033	0,901	0,346	0,174
**Ki-67**	-0,057	0,811	0,382	0,106	0,025	0,918	–0,426	0,078	–0,570	**0,014**
**sTn**	–0,031	0,898	–0,378	0,171	0,457	**0,049**	0,372	0,128	0,286	0,250
**sLe^a^**	0,016	0,945	–0,236	0,346	0,175	0,486	0,471	**0,048**	0,171	0,512
**sLe^x^**	0,140	0,556	–0,027	0,912	0,015	0,950	0,000	1,000	0,679	**0,002**

**Figure 2:**
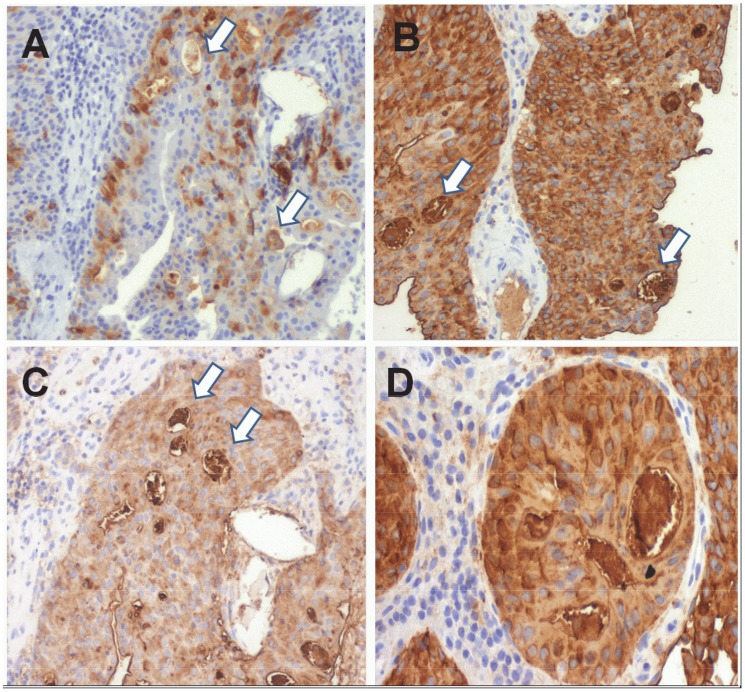
Expression of cancer-associated glycans antigens A) sTn, B)sLea and C-D) sLe^x^ in *Schistosoma haematobium* eggs. The white arrows point to positive eggs. The treatment of the tissue sections with a α-neuraminidase led to the loss of immunoreactivity, confirming the validating the structural assignment.

Moreover, it was observed that 60% of the cases presented *S. haematobium* eggs embedded in the bladder urothelium, predominantly in benign/pre-malignant lesions without the presence of tumour (75% vs 45%). However, no associations were found between the expression of the studied markers and the presence and absence of the eggs in the bladder at the time of diagnosis. Altogether, these findings suggest that the presence of eggs in the bladder may be an early event leading to carcinogenesis. Whether the disorganization of the tissue associated with malignant transformation may favor their release into the environment, therefore explaining is lower presence in malignant tissues and consequently the lack of correlation with the studied biomarkers, warrants further investigation. It was further observed that the majority of the cases (>75%) presented sLe^a^ and sLe^a^ positive eggs and approximately half displayed eggs expressing the sTn antigen, suggesting some degree of mimicry of host glycosylation patterns (Fig. 2). The expression of sialylated glycans was validated by observing the loss of reactivity against anti-glycans monoclonal antibodies after treatment of the tissue with a neuraminidase. It was noteworthy that both positive and negative eggs for these antigens could be found within the same biopsy, denoting some degree of heterogeneity at this level.

## Discussion

In contrast to the extensive cytogenetic and molecular signatures existing for urothelial cell carcinoma, mainly found in western populations, little is known about the molecular alterations underlying the development of *S. haematobium*-associated bladder cancer. Nevertheless, such information is pivotal to support a definitive association between schistosomiasis and bladder cancer development that, until now, has been mostly supported by epidemiological studies. Furthermore, it may provide means for an early identification of infected populations at a risk of developing bladder cancer, which is a particular critical matter since the majority of the cases are detected at a late stage due the absence of appropriate medical facilities.

Herein we have screened a series of schistosome-associated bladder tumours, their adjacent mucosa and also biopsies of apparently normal urothelia, for bladder cancer biomarkers. We also included a series of bladder tumours of non-schistosome etiology and normal urothelium sections in an attempt to highlight common molecular alterations. The majority of the patients enrolled in this study were females, and this is in clear contrast with the higher prevalence of bladder cancer among men in western populations. This may be explained by social and working habits of these populations living in the proximity of contaminated water courses. Also, the majority of the cases of bladder cancer were observed among young adults, which is rare for urothelial carcinomas of chemical etiology [40]. These findings were in accordance with previous observations from other authors [5], [8] and support a role for the parasite in cancer development.

The evaluated biomarkers included the accumulation of oncoprotein p53 [22]–[24] and tumour proliferation index given by Ki-67 overexpression [26]–[28], two events associated with the aggressiveness of urothelial bladder cancer. Likewise, we observed alterations in p53 in the majority of schistosome-associated bladder tumours, irrespectively of their histopathological nature, which is in agreement with our findings and previous observations for non-schistosome associated tumours [25], [41]. In addition, these alterations were not observable in the normal urothelium, denoting its cancer-related nature. The association between the accumulation of p53 in the urothelium and infection with *S. haematobium* reinforces the notion that the parasite may contribute to profound alterations in urothelial cells, ultimately leading to aggressive forms of cancer. This hypothesis is further supported by the observations reported by Botelho and colleagues [8], [19]. According to these authors, the exposure to *S. haematobium* antigens down-regulates cell apoptotic pathways, which would ultimately lead to the development of cancer [42]. Regarding proliferation, Ki-67 overexpression was lower in low malignant potential lesions when compared to urothelial and squamous cell carcinomas. This is in accordance with previous findings associating the degree of severity of bladder malignant lesions with higher proliferation degrees and potential to evolve to more aggressive forms of cancer [26], [31]. Since several markers were evaluated and a large numbers of comparisons were performed, the false discovery rate should be considered. Nevertheless, if all the null hypotheses are true, 5% of the comparisons are expected to present uncorrected *P* values lower than 0.05 by chance alone. However, our study present *P* values under 0.05 in 20% of the comparisons, thereby demonstrating the statistic value of the observations.

The expression of cancer-associated cell-surface sialylated glycans sTn, sLe^a^ and sLe^x^ was addressed, to our knowledge, for the first time in *S. haematobium-*associated bladder tumours. Glycosylation is the main and more complex posttranslational modification of membrane-bound and secreted proteins. Glycans plays a key role in protein folding and stability [43], mediate several physiological and pathological conditions, which include cell-cell adhesion, host-pathogen interactions, cell differentiation, migration and cell trafficking, signaling and immune recognition [44], [45]. During malignant transformation, some cells change their glycosylation profile in response to microenvironment challenge, namely paracrine signaling, hypoxia among other events [29]. The sTn antigen, resulting from a premature stop in the *O*-glycosylation of proteins by sialylation, has been found associated with high-grade bladder non-muscle invasive papillary tumours and muscle invasive lesions [30], [31]. It has been found to enhance bladder cancer cells capability to invade and migrate [31] and acts as a suppressor of effective dendritic cell immune responses against bladder cancer cells [46]. Despite the low number of cases, this study suggests that the sTn antigen is predominantly expressed by more aggressive forms of *S. haematiobum* associated tumours (UCC+SCC). These observations are in accordance with our previous results from non-schistosome associated bladder tumors, were sTn expression is predominant found in high grade papillary tumors and muscle invasive bladder cancer of non-schistosome etiology [31]. The sialylated Lewis blood group determinants sLe^a^ and sLe^x^ may be found as terminal structures of both proteins and lipids and have been associated with metastatic potential and poor overall survival in several solid tumours [47]–[50]. The sLe^a^ antigen has also been found both in pre-malignant bladder lesions, non-invasive and invasive bladder urothelial carcinomas [32]–[34]; however no association with recurrence, invasion or metastasis has been reported. On the other hand, its structural isomer sLe^x^ antigen was observed in muscle invasive urothelial carcinomas associated with invasion, metastasis and recurrence [35], [36]. This study now demonstrates that the majority of schistosome-associated bladder tumours expressed the sLe^x^ antigen, suggesting a high degree of malignant potential. However, no defined expression pattern could be drawn for sLe^a^ Similarly, we also found significant overexpression of the sLe^x^ antigen in non-schistosome associated tumours but also a more pronounced expression of sLe^a^. However, none of these antigens were not observed in the healthy urothelium, reinforcing its malignant nature.

The analysis of benign/pre-malignant lesions, irrespectively of their origin, showed a predominance of p53, sLe^a^ and sLe^x^ positive cases. Noteworthy, sole lesions were predominantly non-proliferative when compared to lesions in the vicinity of tumours, suggesting that high proliferation may be mainly a characteristic of the tumour. Whether proliferative benign/pre-malignant lesions present a higher risk of evolving to bladder cancer warrants validation in future studies.

On the other hand, the sLe^a^ antigen was predominantly expressed among sole lesions, denoting this antigen may constitute a marker of early bladder carcinogenesis mediated by *S. haematobium*. Reinforcing this observations, Kajiwara et al. has described that sLe^a^ is inversely associated with the grade of atypia while its non-sialylated form DU-PAN-2 correlates with the grade of atypia in urothelial carcinomas; these authors also observed that the disappearance of sLe^a^ and the presence of DU-PAN-2 correlates with high malignant potential [36]. We further observed that the expression of cancer-associated antigens in the tumour was correlated with the expression denoting a field effect that affects the entire bladder. Again, this correlation was not observed for proliferation, reinforcing this event see ms to be mainly a characteristic of malignant lesions. Taken together, these observations highlight that pre-malignant lesions present molecular alterations associated with malignancy, and that p53 and sLe^x^ are surrogate markers of bladder cancerization associated with infection with schistosomes. More studies should be conducted to validate the potential of sLe^a^ has a surrogate marker of infection that may be helpful in the monitoring of asymptomatic colonization. A glycoproteomic/lipidomic characterization of bladder tumours is ongoing, which is expected to provide their necessary insights about the biologic role of the studied glycans in bladder cancer.

These observations are also likely to be of consequence in the clinic of great importance since benign and pre-malignant lesions such as those included in this study are challenging to diagnose by cystoscopy. We emphasize the potential of glycans in context, as they can be found at the cell-surface, thus easily accessible to antibodies and other carbohydrate ligands and consequently be explored in cancer detection imaging [51], [52]. They are often secreted into the blood stream and urine and therefore readily accessible in non-invasive diagnosis [34], [37], [38], [53]. Non-invasive diagnostic procedures are critical as they facilitate large scale screening of the populations in endemic regions where imaging/radiological facilities are not likely to be available.

Glycans are also important mediators in the colonization of humans by parasites, as they provide means for efficient adhesion and immune escape [54], [55]. As such, we have also addressed the expression of cancer-associated glycans in eggs of *S. haematobium* embedded in the bladders. We observed, for the first time, that the parasite eggs express sLe^a^ and sLe^x^ antigens, in mimicry of human leukocytes. These glycans are specific ligands for E-selectin, a cell adhesion molecule expressed only on endothelial cells and activated by cytokines, such as IL-1 and TNF-α, released by damaged cells during the course of inflammation [56], [57]. Cytokines induce the overexpression of E-selectin by endothelial cells on nearby blood vessels that are responsible for recruiting leukocytes in a sLe^a^/sLe^x^-mediated manner [56], [57]. These glycans bind weakly to E-selectin which allows leukocytes to “roll” along the internal surface of the blood vessel into the injury site by shear forces of blood flow [58]. Similar events may drive the recruitment of *S. haematobium* eggs to the bladder wall, a critical step in the developmental cycle of this pathogen. Similar strategies have been observed in nature, namely by the Gram-negative *Porphyromonas gingivalis* to adhere to human umbilical vein endothelial cells [59]. Several authors have also hypothesized that E-selectin-sialylated glycans interactions may contribute to the hematogenous dissemination of sLe^a^/sLe^x^ expressing tumour cells and explain its association with metastasis [29], [47], [49], [60]. Similarly, for bladder tumors, the identification of the parasite glycoproteins and/or glycolipids presenting these alterations may bring insights on this matter and ultimately contribute to design strategies to control infection. In addition, the identification of the glycoproteins and/or glycolipids presenting these alterations may yield insights into this infection-associated cancer and ultimately contribute to design strategies to control infection. Glycoproteomic studies will greatly benefit from the recent mapping of the parasite genome [61]. We further report that some eggs express the sTn antigen, an oncofetal antigen that, we and others have shown to play a key role in immune escape [46]. The sLe^x^ expression has also been found to reduce the susceptibility of tumour cells to hepatic sinusoidal lymphocyte-mediated killing, and thus, may facilitate the ability of the tumor cells to metastasize to the liver [62]. Similarly, the expression of these glycans by *S. haematobium* may provide the necessary means for immune escape either by modulation of the immune system or by molecular mimicry of the host, a common survival strategy among parasites [63], [64].

To conclude p53 and sialylated Lewis blood group determinants may be surrogate markers of cancerization associated with chronic infection with *S. haematobium*. By drawing attention to common molecular pathways underlying these two events, this study provides one of the missing links associating parasite infection and cancer development. Further studies which include a larger sample of *Schistosoma haematobium* positive cases will be needed to determine a panel of biomarkers with the potential to identify bladder cancer precursor lesions Finally, this report provides insights on the glycosylation patterns of *S. haematobium* eggs and discusses a possible model for the recruitment of eggs to the bladder wall that suggests this schistosome has evolved glycosylation patterns that mimic those of its human host. These insights may help guiding the development of novel therapeutic strategy, namely glycoconjugate vaccines.
